# Biocompatibility of *Synechococcus* sp. PCC 7002 with Human Dermal Cells In Vitro

**DOI:** 10.3390/ijms25073922

**Published:** 2024-03-31

**Authors:** Benedikt Fuchs, Sinan Mert, Constanze Kuhlmann, Sara Taha, Alexandra Birt, Jörg Nickelsen, Thilo Ludwig Schenck, Riccardo Enzo Giunta, Paul Severin Wiggenhauser, Nicholas Moellhoff

**Affiliations:** 1 Division of Hand, Plastic and Aesthetic Surgery, LMU University Hospital, LMU Munich, 80336 Munich, Germany; sinan.mert@med.uni-muenchen.de (S.M.); constanze.kuhlmann@med.uni-muenchen.de (C.K.); sara.taha@med.uni-muenchen.de (S.T.); alexandra.birt@med.uni-muenchen.de (A.B.); praxis@drschenck.com (T.L.S.); riccardo.giunta@med.uni-muenchen.de (R.E.G.); severin.wiggenhauser@med.uni-muenchen.de (P.S.W.); nicholas.moellhoff@med.uni-muenchen.de (N.M.); 2 Molecular Plant Science, Department Biology I, LMU Munich, 80336 Munich, Germany; joerg.nickelsen@lrz.uni-muenchen.de

**Keywords:** *Synechococcus* sp. PCC 7002, cyanobacteria, coculture, fibroblasts, keratinocytes, biocompatibility

## Abstract

Being the green gold of the future, cyanobacteria have recently attracted considerable interest worldwide. This study investigates the adaptability and biocompatibility of the cyanobacterial strain *Synechococcus* sp. PCC 7002 with human dermal cells, focusing on its potential application in biomedical contexts. First, we investigated the adaptability of *Synechococcus* PCC 7002 bacteria to human cell culture conditions. Next, we evaluated the biocompatibility of cyanobacteria with common dermal cells, like 3T3 fibroblasts and HaCaT keratinocytes. Therefore, cells were directly and indirectly cocultured with the corresponding cells, and we measured metabolic activity (AlamarBlue assay) and proliferation (cell count and PicoGreen assay). The lactate dehydrogenase (LDH) assay was performed to determine the cytotoxic effect of cyanobacteria and their nutrition medium on human dermal cells. The cyanobacteria exhibited exponential growth under conventional human cell culture conditions, with the temperature and medium composition not affecting their viability. In addition, the effect of illumination on the proliferation capacity was investigated, showing a significant impact of light exposure on bacterial growth. The measured oxygen production under hypoxic conditions demonstrated a sufficient oxygen supply for further tissue engineering approaches depending on the number of bacteria. There were no significant adverse effects on human cell viability and growth under coculture conditions, whereas the LDH assay assessed signs of cytotoxicity regarding 3T3 fibroblasts after 2 days of coculturing. These negative effects were dismissed after 4 days. The findings highlight the potential of *Synechococcus* sp. PCC 7002 for integration into biomedical approaches. We found no cytotoxicity of cyanobacteria on 3T3 fibroblasts and HaCaT keratinocytes, thus paving the way for further in vivo studies to assess long-term effects and systemic reactions.

## 1. Introduction

In contemporary society, there is an observable trend towards the use of nature-derived products [1,2]. Being the green gold of the future, microalgae and cyanobacteria have recently attracted considerable interest worldwide [3,4]. Their metabolites, such as lipids, protein, pigments, and bioactive compounds, have immense potential for sustainable energy and pharmaceutical production capabilities [5,6,7]. This development has also permeated the field of medicine, where plant organisms are being increasingly used in regenerative medicine. The scientific community has recently placed particular emphasis on cyanobacteria, which, owing to their positive attributes, qualify as an ideal microorganism for tissue engineering [8,9]. Cyanobacteria are photosynthetic prokaryotic organisms. They occupy fresh, brackish, and marine waters around the world [10]. These microorganisms are among the primary producers of environmental oxygen [11]. These organisms could promote a local and controllable source of oxygen to circumvent the need for blood perfusion to sustain tissue survival. After genetic modification, they have high potential to deliver recombinant growth factors to incisional wounds.

The new popularity in their medical application is based on their various advantages over other microorganisms. These organisms offer resistance to external influences, a capacity for photosynthetic oxygen production, and accessibility to genetic manipulation [12]. They offer an autotrophic microorganism-based research platform with unique advantages as “autotrophic cell factories” [13,14]. For example, cyanobacteria were developed as a “green” chassis for the synthesis of bioproducts [14,15]. They directly use CO_2_, light energy, and inorganic nutrients to synthesize versatile plant-specific photosynthetic intermediates and organic compounds in large-scale photobioreactors with outstanding economic merit [16]. Further cyanobacteria were engineered for the production of the bulk chemical butanol, specifically isobutanol and 1-butanol [17,18], and several biomaterials, like spider silk [7] and glycosaminoglycans [19].

Their photosynthetic capacity allows a local oxygen supply independently of the surrounding tissue. Their implementation as a strategic approach to locally increase the oxygen supply was shown to be effective in skin, pancreas, heart, brain, and tumor tissues [8,20,21]. These unique properties enable, for example, a photosynthetic symbiotic therapy to rescue the ischemic myocardium, utilizing the cyanobacterium *Synechococcus elongatus* to metabolize cardiomyocyte-derived carbon dioxide and release new oxygen for sustained aerobic metabolism during ischemia [22].

In recent decades, efforts have been made to enhance the usage of microalgae and cyanobacteria through genetic manipulation and synthetic and metabolic engineering [23,24]. Due to the light dependency of the bacteria, we have seen the breakthrough of the bacteria primarily in the area of the body surface, such as a dermal application [25]. Our research group aims to enhance the treatment of chronic wounds by seeding these bacteria in collagen-based scaffolds [26,27]. The photosynthetic oxygen production facilitates overcoming the hypoxic wound environment, while genetic modification allows the release of deficient growth factors into the wound bed [28]. A specific focus has been placed on stimulating lymphangiogenesis through bacterially secreted hyaluronic acid, aiming to improve lymphatic drainage and reduce inflammation. Thus, cyanobacteria exhibit significant potential for advancing the regeneration of chronic wounds [26].

Prior studies have either been conducted using animal models or have focused on human interstitial epithelial cells. Our research group has successfully developed a cyanobacteria-bioactivated scaffold and examined its regenerative potential in vitro. The next phase involves a dermal application, necessitating an assessment of the initial influence of *Synechococcus* sp. PCC 7002 on human dermal cells. Consequently, it is of paramount importance to rule out the cytotoxic effects of the bacteria and their cultivation media conclusively. However, there are few studies investigating the direct impact of *Synechococcus* sp. PCC 7002 on human dermal cells in the literature. Therefore, further research projects demand a more intensive evaluation of cellular coexistence. For this purpose, cyanobacteria were cocultured with the most common dermal cells, such as fibroblasts and keratinocytes.

In summary, cyanobacteria offer many advantages leading to their increasing popularity in biomedicine. In this study, we investigate the biocompatibility of cyanobacteria with the classical representatives of epidermal and dermal cells, in particular fibroblasts and keratinocytes. We hypothesized that cyanobacteria can be cultured under human cell culture conditions without significant growth restrictions. Secondly, we hypothesized that the proliferation and survival of human dermal cells are not significantly affected by being cocultured with cyanobacteria.

## 2. Results

### 2.1. Adaptation of Cyanobacteria to Human Cell Culture Conditions

**Figure 1:**
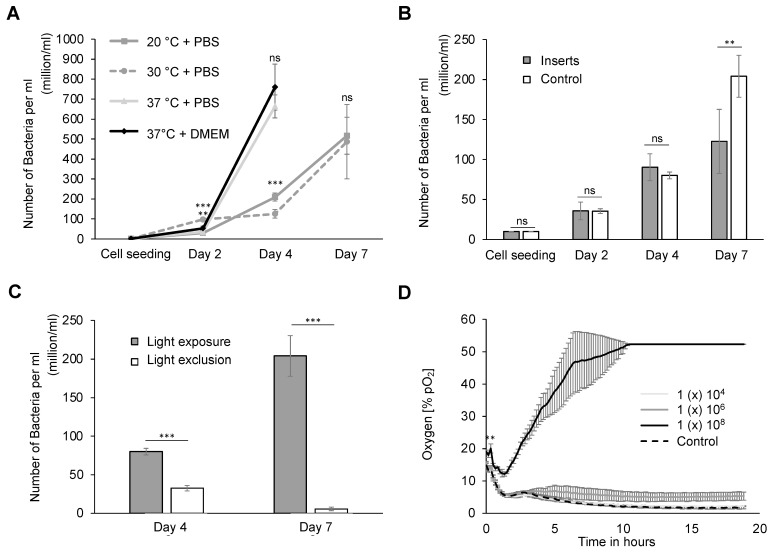
Adaptation of cyanobacteria to human cell culture conditions. (**A**) Evaluation of the proliferation capacity of *Synechococcus* sp. PCC 7002 through cell counting in the Neubauer cell chamber at different temperatures and time points. The bacterial population was assessed at 20 °C after 2 days 29.91 × 10^6^ (±2.18 × 10^6^), 4 days 209.00 × 10^6^ (±19.73 × 10^6^), and 7 days 516.67 × 10^6^ (±92.51 × 10^6^); at 30 °C after 2 days 97.33 × 10^6^ (±8.96 × 10^6^), 4 days 125.67 × 10^6^ (±21.22 × 10^6^), and 7 days 48.00 × 10^6^ (±18.5 × 10^6^); and at 37 °C with the A-D7 medium + PBS after 2 days 34.96 × 10^6^ (±5.9 × 10^6^) and 4 days 663.33 × 10^6^ (±57.73 × 10^6^); and at 37 °C with the A-D7 medium + DMEM after 2 days 52.88 × 10^6^ (±11.21 × 10^6^) and 4 days 760 × 10^6^ (±115.32 × 10^6^) (*n* = 3). (**B**) The use of cell culture inserts (0.4 µm pore size) compared to a positive control. The quantification of bacterial proliferation was performed in 12-well plates with either cell culture inserts (0.4 µm pore size) or a positive control. In the control group, a bacterial count of 35.60 × 10^6^ (±11.01 × 10^6^) was observed after seeding 1 × 106 bacteria, with subsequent counts of 90.00 × 10^6^ (±16.8 × 10^6^) after 4 days and 204.00 × 10^6^ (±26.23 × 10^6^) after 7 days. When bacteria were cultivated in cell culture inserts, counts of 35.33 × 10^6^ (±3.05 × 10^6^) were recorded after one day, 80.00 × 10^6^ (±9.24 × 10^6^) after 4 days, and 122.00 × 10^6^ (±40 × 10^6^) after 7 days (n = 3). (**C**) Assessment of bacterial proliferation under varying light conditions. The determination of bacterial proliferation involved cell counting under constant illumination and light exclusion. After light exposure, counts on day 4 were 80.00 ± 4.24 × 10^6^, and on day 7 were 204.00 ± 26.23 × 10^6^. In the absence of light, counts on day 4 were 32.50 ± 3.53 × 10^6^, and on day 7 were 5.66 ± 2.31 × 10^6^. (**D**) Measurement of oxygen concentrations in the culture medium. Oxygen concentrations in the culture medium were measured using the SensorDishReader (SDR; Presens GmbH, Regensburg, Germany) at 10 min intervals over a 20 h period. The photosynthetically produced oxygen was investigated at the bacterial concentrations of 1 × 10^4^, 1 × 10^6^, and 1 × 10^8^ (n = 3). All experiments were performed in triplicate, and statistical analysis was conducted using an unpaired Student’s *t*-test, denoted as ns (not significant), ** *p* < 0.01, and *** *p* < 0.001.

Initially, an assessment of the growth and oxygen production of the cyanobacteria *Synechococcus* sp. PCC 7002 was performed under conventional human cell culture conditions to ascertain their viability within parameters established for human systems (37 °C, 5% CO_2_). The bacteria were cultivated and compared in both the standard A-D7 medium and a 1:1 dilution of a bacterial medium with the cell types’ respective culture media (Dulbecco′s modified Eagle′s medium, DMEM). The initial investigation focused on the impact of the temperature and medium composition on bacterial viability. Bacterial counts, as determined in the Neubauer cell chamber, revealed exponential growth after 2 and 4 days at 37 °C (Figure 1A). Noteworthy differences in bacterial numbers between those cultivated in the A-D7 medium supplemented 1:1 with DMEM or phosphate-buffered saline (PBS) were only observed on day 2. Additionally, a flattened growth curve due to a deviation from the optimum temperature (37 °C) was identified. Cyanobacterial growth was markedly reduced at both 20 °C and 30 °C in the initial 4 days. Day seven revealed increased bacterial numbers after a week, indicating vitality despite a reduced growth rate. 

The impact of cultivating cyanobacteria in cell culture inserts on bacterial growth was investigated in preparation for cocultures with human cells (Figure 1B). Cell culture inserts permit the spatial separation of two cultures while facilitating medium and nutrient exchange through a 0.4 µm pore membrane. In the initial 4 days, bacterial counts exhibited exponential growth without significant differences when inserts were used compared to the positive control (bacterial cultivation in regular wells). After 7 days, however, a twofold higher cell count of 204 ± 26 million was observed with regularly cultivated bacteria in a 12-well plate compared to the bacteria in cell culture inserts (122 ± 40 × 10^6^), possibly due to the reduced cultivation area in the inserts, associated with a restriction of proliferation. Furthermore, the dependence of bacterial growth on light exposure was examined (Figure 1C). Constant illumination at a distance of 25 cm resulted in an exponential increase in the bacterial count to 204 ± 26 million within 7 days. Conversely, the exclusion of light led to a reduction in the number of bacteria to 5.7 ± 2.3 million, highlighting the bacteria’s reliance on light exposure, necessitating a light source for subsequent experiments. The photosynthetic properties of cyanobacteria allowed the measurement of oxygen production under constant light stimulation (Figure 1D). To simulate hypoxic conditions, which are characteristic of chronic wounds, the experiment was performed under oxygen deprivation (<1% O_2_). Under hypoxic conditions, the synthesized oxygen exhibited dependence on the bacterial seeding concentration, reaching an oxygen content of over 50% after 10 h at a bacterial concentration of 1 × 10^8^/mL. Concentrations lower than 1 × 10^6^ bacteria per ml yielded low oxygen concentrations of 5.97 ± 1.25% at a bacterial number of 1 × 10^6^ and 3.62 ± 0.21% at a bacterial number of 1 × 10^4^ on average. A bacterial count of at least 1 × 10^7^ was, therefore, used for all subsequent experiments to ensure sufficient oxygenation. In summary, the culture conditions of *Synechococcus* sp. PCC 7002 can be adapted to human cell conditions at 37 °C, with the supplementation of human cell media and using inserts under the conditions of constant illumination. 

### 2.2. Assessment of Cyanobacterial Biocompatibility with 3T3 Fibroblasts

**Figure 2:**
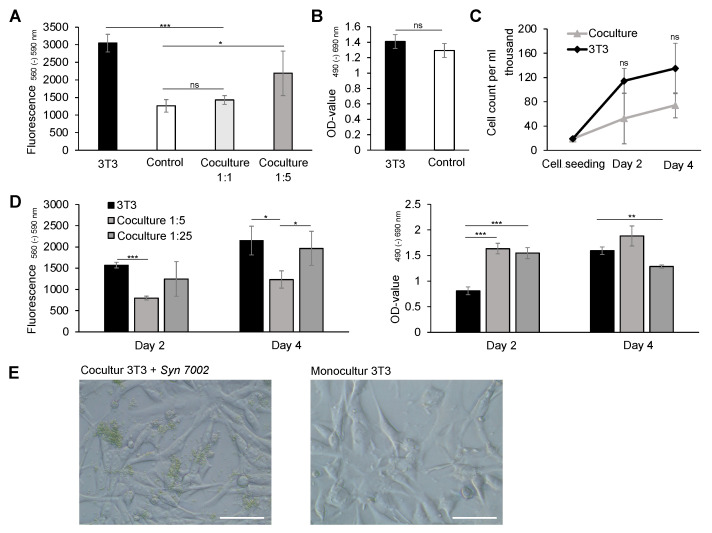
Assessment of the biocompatibility of 3T3 fibroblasts with Syn 7002. (**A**) Biocompatibility was assessed by determining the metabolic activity using the AlamarBlue assay after a 4-day incubation period. Fluorescence signals were measured at an excitation wavelength of 560 nm and an emission wavelength of 590 nm. Results are expressed as the mean ± standard deviation: 3T3: 3046.67 ± 251.38; control: 1261.67 ± 178.6; coculture 1:1: 1429.34 ± 123.54; and coculture 1:5: 2187.67 ± 633.1. (**B**) The cytotoxicity of the bacteria-specific A-D7 medium on 3T3 fibroblasts was evaluated using the lactate dehydrogenase (LDH) assay in a 3T3 culture after 4 days. The treatment group received 1:1 diluted A-D7 medium, whereas the control received pure DMEM medium. Absorbance was measured at a wavelength of 490 nm with a reference wavelength of 690 nm. OD values: 3T3: 1.4 ± 0.09; and control: 1.29 ± 0.89. (**C**) Cell count analysis, using Counts FL2, was performed to detect a reduced growth rate, resulting from a 1:1 dilution of the DMEM cell medium with A-D7 in bacterial cocultures compared to a 3T3 pure culture over a 4-day period. Cell count per ml after 2 days: 3T3: 11.43 ± 20.71 × 10^6^; coculture: 52.55 ± 41.79 × 10^6^; and after 4 days: 3T3: 134.96 ± 41.49 × 10^6^; coculture: 74.26 ± 20.56 × 10^6^. (**D**) Left: Metabolic activity and cytotoxicity assessment: The AlamarBlue assay was used to determine the metabolic activity and cell survival of 3T3 fibroblasts in cocultures with Syn 7002. Fluorescence signals were measured at an excitation wavelength of 560 nm and an emission wavelength of 590 nm after 2 and 4 days. Right: Additionally, we assessed the cytotoxicity of cyanobacteria on 3T3 fibroblasts by the lactate dehydrogenase assay (LDH). Absorbance was measured at a wavelength of 490 nm with a reference wavelength of 690 nm. (**E**) Microscopic evaluation was performed at 20× magnification to visually represent a coculture of 3T3 fibroblasts with *Synechococcus* sp. PCC 7002 (Syn 7002) at a ratio of 1:5 (**left**) compared to a 3T3 monoculture of a control group (**right**). Scale bar: 200 µm. The study included 10 replicates for the coculture (n = 10) and 12 replicates for the monoculture (n = 12). ns > 0.05, * *p* < 0.1, ** *p* < 0.01, and *** *p* < 0.001.

To investigate the biocompatibility of cyanobacteria with 3T3 fibroblasts in vitro, the cocultivation was examined under standard cell culture conditions. The initial phase involved assessing the potentially toxic effects of cyanobacteria on 3T3 fibroblasts through an indirect coculture system using cell culture inserts with a permeable membrane (0.4 µm pore size). This configuration facilitated physical separation, while allowing the exchange of cultural media. The cocultures were maintained under constant illumination for 2 and 4 days at 37 °C and 5% CO_2_. After a single measurement after 4 days, we observed a notable enhancement in the metabolic activity by the AlamarBlue assay when 3T3 fibroblasts were cocultured with a 5-fold higher bacterial concentration, exhibiting a 1.5-fold higher fluorescence signal in 3T3 cell activity (OD values of 2187.6 ± 633.09) compared to a 1:1 bacterial ratio (Figure 2A). A statistical significance was observed relative to the control with the absence of aquacultures. Furthermore, we assessed a 2.5-fold higher metabolic activity in the 3T3 culture with the cell-specific DMEM compared to the control with a 1:1 diluted DMEM in a bacteria-specific A-D7 medium. To investigate any toxic effects of the bacteria’s A-D7 medium on 3T3 fibroblasts, an LDH assay was performed, indicating no significant differences in the activity of lactate dehydrogenase between the media (Figure 2B). Next, we observed that a 1:1 dilution resulted in a halving of the cell activity. This observation was confirmed in a cell count of 3T3 fibroblasts, although not statistically significant, indicating that cocultures supplemented with bacteria-specific A-D7 medium suffer a growth disadvantage with an approximately 50% reduction in proliferation (Figure 2C). After 2 days of cocultivation, 3T3 fibroblasts reached a cell count of 52.55 ± 41.79 × 10^6^ and after 4 days 74.26 ± 20.56 × 10^6^ compared to fibroblasts without bacterial medium supplementation. 

Subsequently, direct cultivation, involving cell–cell contact between both organisms, was examined. The AlamarBlue assay demonstrated a reduced metabolic activity in cocultures after 2 and 4 days (Figure 2D, left). Due to the observed advantage of a higher bacterial count on the metabolic activity of fibroblasts in the initial indirect cocultures, the bacterial count was increased to 25 times that of the 3T3 cell count in the experimental setup. We noted a significant increase in cocultures with a 25-fold bacterial increase compared to a 1:5 ratio with the bacteria. The cells responded with a markedly enhanced activity to the increase in the bacterial count. To investigate the potential cell apoptosis over the 4-day period of cocultivation, we detected lactate dehydrogenase in the cell medium (Figure 2D, right). In the 3T3 monoculture, a doubling of the LDH content was measured, whereas in cocultures at a 1:25 ratio, a reduction in lactate dehydrogenase was observed. Cocultures at a 1:5 ratio showed only a slight increase in LDH concentration from 1.03 ± 0.1 to 1.88 ± 0.19 after 4 days. Accordingly, lactate dehydrogenase measurements indicated bacterial cytotoxicity after 2 days. However, the LDH trend after 4 days suggests no cytotoxicity in bacterial cultures. Microscopic images confirmed the coexistence of both organisms after 4 days, providing evidence of biocompatibility (Figure 2E).

### 2.3. Biocompatibility of HaCaT Keratinocytes and Cyanobacteria

**Figure 3:**
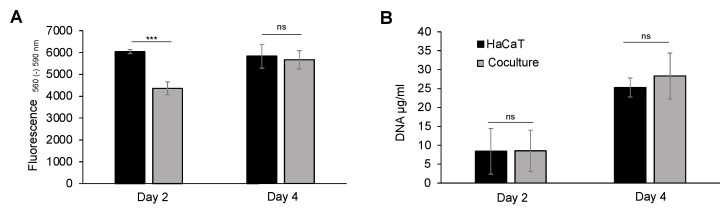
Assessment of HaCaT keratinocytes’ biocompatibility with Syn 7002. (**A**) Metabolic activity of HaCaT keratinocytes was determined using the AlamarBlue assay to detect cell survival in a coculture with cyanobacteria. Fluorescence signals were measured at an excitation wavelength of 560 nm and an emission wavelength of 590 nm. After 2 days, we assessed the fluorescence intensity of HaCaT: 6045 ± 92; coculture: 4360.33 ± 297; and after 4 days: HaCaT: 5831.66 ± 545.08; coculture: 5670.33 ± 415.70. (n = 3). (**B**) Proliferation of HaCaT keratinocytes in a coculture with Syn 7002 over a 4-day period was assessed using the PicoGreen assay, measuring the DNA concentration in µg/mL. After 2 days: HaCaT: 8.55 ± 2.52 µg/mL; coculture: 8.41 ± 6.03 µg/mL; coculture; and after 4 days: HaCaT: 28.31 ± 6.06 µg/mL; coculture: 25.29 ± 5.44 µg/mL (*n* = 3). All experiments were performed in triplicate, and an unpaired Student’s *t*-test was conducted, indicating non-significance (ns). ns > 0.05 and *** *p* < 0.001.

Keratinocytes, at varying stages of keratinization, are integral to the architectural composition of the stratified epidermis and, consequently, serve as the immediate interface between the body and its external milieu. It is, therefore, of particular interest to evaluate the compatibility of the bacterial suspensions with HaCaT keratinocytes. Acknowledging the previously noted deceleration in the growth rate of 3T3 fibroblasts attributable to dilution, this phenomenon was systematically considered in the subsequent keratinocyte controls. The preparation of media for coculture closely mirrored the dilution conditions. In the pursuit of establishing biocompatibility, a direct coculture was instituted, wherein the two cell populations were not segregated, and metabolic activity was quantified using the AlamarBlue assay (Figure 3A). Evidently, there was a discernible increase in the metabolic activity within the coculture culminating in the absence of statistically significant disparities between the fluorescence intensity of the coculture (5670.33 ± 415.70) and the monoculture of keratinocytes (5831.66 ± 545.08) after a 4-day duration. Concurrently, the control group exhibited a consistent metabolic activity over the same time span. Due to the detection of a markedly higher metabolic activity in the control group after 2 days, suggesting a plausible incongruity between the two cell populations, an investigation into cell proliferation was performed. Therefore, we assessed the DNA content with a PicoGreen assay (Figure 3B). The results reveal no significant differences both at the 2-day and 4-day intervals. Notably, there was a consistent increase in the DNA concentration over the 4-day period, correlating with metabolic activity increments in both experimental groups. Consequently, the findings do not indicate any observable toxic effects of cyanobacteria on human keratinocytes, thereby suggesting an in vitro biocompatible interaction between the two organisms.

## 3. Discussion

Plant-based products are becoming increasingly important in skin regeneration. In general, a trend towards natural ingredients and nature-based products (such as creams and peelings) can be observed in society [1]. Recently, algae and bacteria have also come increasingly into focus for tissue regeneration in medicine. As algae have already proven to be compatible with humans, it is of particular interest to test the effect of cyanobacteria on human dermal cells. The biocompatibility of cyanobacteria with human dermal cells enables a broad application in regenerative biomedicine [20]. Previous investigations have primarily focused on the effects of individual toxins or extracts from *Synechococcus* strains on the skin, with secondary metabolites being employed in natural anti-aging cosmetics [29]. No studies have investigated the direct cell–cell contact of cyanobacteria with mammalian blood cells. To date, only bacterial toxins have been applied to mammalian cells, showing no adverse effects on the surrounding tissue. Morone et al. evaluated the potential of acetonic and aqueous extracts from cyanobacteria strains on human dermal cells for cosmetic application, showing no cytotoxic effects [25]. Incompletely, only selected proteins were tested in this analysis, and possible cell–cell interactions were excluded. 

However, we advocate for a direct application of these microorganisms to achieve long-term and localized effects, fully exploiting the entire potential of cyanobacteria. The skin, along with contaminated wounds, is already inhabited by microflora [30,31]. Thus, supplementing pro-regenerative microorganisms and harnessing them through a photosymbiotic approach seems obvious. The interplay between bacteria and human cells is vital in various areas of the body [32,33,34]. In the context of the skin, a successful symbiosis at the molecular level could enhance wound healing. 

Cyanobacteria are an increasingly popular microorganism due to their properties for the photosynthetic production of oxygen. For instance, the application of oxygen-producing cyanobacteria in tissue engineering for treating chronic wounds, characterized by bacterial miscolonization, hypoxia in the wound bed, and a local growth factor deficiency, holds promise [35]. Additionally, they are simply genetically modified for the synthesis of growth factors that are crucial for skin regeneration by secreting them onto the skin and into the wound bed, addressing local growth factor deficiencies [26]. The potential applications of cyanobacteria span from the in vitro creation of artificial human tissues to the photosynthetic maintenance of oxygen-deprived organs in both in vivo and ex vivo settings, with an ongoing exploration of their broader medical utility [20]. The advantages of incorporating *Synechococcus* sp. strain PCC 7002 in this study and its relevance in the biomedical domain for crafting materials intended for skin regeneration are elucidated in this paper. Relative to the microalga *Chlamydomonas reinhardtii*, hitherto used by Schenck et al. [36], unicellular euryhaline, mixotrophic cyanobacteria present a myriad of advantages for cutaneous applications within inflammatory tissues and experimental manipulations. Noteworthy is the proliferative prowess of *Synechococcus* sp. PCC 7002, exhibiting an average doubling time of 4 h—twice as rapid as the growth exhibited by *Chlamydomonas reinhardtii*, which has an approximate doubling time of 8–10 h [37]. 

In our study, we first investigated the prerequisites governing the use of cyanobacteria in medical and human contexts. We assessed whether cyanobacteria could be cultivated under human cell conditions. The cultivation conditions of human cells differ from those of bacteria due to a lack of rotation of the liquid culture (150 rpm), lack of light exposure, reduced temperature and increased CO_2_ concentration, and altered medium composition. Therefore, it is particularly important to test whether the cyanobacteria can withstand adaptation to human conditions. Furthermore, it is imperative to consider the exclusion of possible cytotoxic effects induced by the culture medium utilized for human cells, specifically Dulbecco’s modified Eagle medium (DMEM), upon cyanobacterial organisms. On top of that, we evaluated the efficacy of using cell culture inserts for prospective investigations. 

The optimum growth temperature for the *Synechococcus* sp. strain PCC 7002 is 38 °C at 1% CO_2_ atmosphere [38], congruent with human physiological conditions, thus facilitating cultivation alongside human dermal cells. We detected a significant reduction in the bacterial growth rate when cultured at 20 °C and 30 °C. There was no restriction of proliferation when adapted to human cell conditions, which included a temperature reduction of 1 degree and an increase in the CO_2_ content to 5%. Additionally, the suboptimal growth temperature for cyanobacteria, maintained below 30 °C (ranging from 18 °C to 28 °C), poses a formidable impediment to coculture production with dermal cells and imposes constraints on proliferation in human dermal applications [39]. Notably, the body temperature on the extremities and acres of the skin typically deviates from the standard core body temperature of 37 °C, contingent upon ambient temperature [40]. Based on the study of Gethin et al., the average wound bed temperature is within the range of 30.2–33.0 °C [41]. Consequently, a commensurate reduction in the proliferation capacity during the dermal application of the bacteria is to be anticipated. Furthermore, we assessed no decrease in proliferation when the bacterial suspension was diluted 1:1 with a regular culture medium (DMEM) instead of PBS, suggesting no toxic effects. As illustrated in Figure 1A,B, a substantial bacterial growth is observed over a period of seven days. Given the significant growth over 4 days and the absence of significance in the subsequent study phases, a toxic effect of the DMEM cell medium on bacterial cultures was dismissed (Figure 1A). 

Next, we evaluated the dependence of the proliferation rate on a continuous light source. We found an exponential increase in the cell number during light exposition compared to cell apoptosis in darkness (Figure 1C). After only 4 days, the number of cells was halved in the absence of a light source. Due to the necessary illumination for the induction of photosynthesis, the resistance of bacteria to high light intensities is of particular importance [42]. Noteworthy is the robust tolerance exhibited by these mixotrophic cultures to extracellular variations, including pH fluctuations, salinity variations, and alterations in the culture medium, with only marginal alterations in the transcriptome pattern [38]. Temperature fluctuations similarly manifest negligible effects on the transcriptome, thereby fortifying the adaptability to oxidative stress—an attribute conducive to surviving in the microenvironment of inflammation zones in chronic wounds, for example [38].

As the bacteria are primarily intended for use in the treatment of chronic wounds, we adapted the experiments accordingly and simulated the low-perfused wound bed using hypoxia. Surprisingly, the oxygen production was strongly dependent on the bacterial concentration. The bacterial concentration of 1 × 10^8^ revealed a high oxygen production after 10 h by using the regular light system for photosynthesis (Figure 1D). This represents a possible independent and constant source of oxygen to oxygenate chronic wounds for regeneration. In the context of this investigation, the adoption of cell culture inserts for cocultivation was paramount, facilitating the meticulous segregation of both organisms devoid of bacterial residues and contaminants. However, a drawback associated with this methodology results in a consequential reduction in the cultivation area (24 wells: 1.9 cm^2^; insert: 0.336 cm^2^) by a factor of approximately 6, thereby evincing a curtailed bacterial proliferation (Figure 1B). In conclusion, the fundamental prerequisites for the use of cyanobacteria within human cellular conditions are delineated in this paper. In our experiments, we successfully demonstrated the cultivation of cyanobacteria under conditions mimicking human cell cultures. This involved adjusting the CO_2_ concentration and temperature, while eliminating the use of a shaking plate. No growth deficit was observed in the bacterial culture maintained at an incubation temperature of 37 °C under a 5% CO_2_ atmosphere. The application of the DMEM cell culture medium did not yield discernible cytotoxic effects, thus affirming the capability of cyanobacteria to thrive under human cell culture conditions. Nevertheless, a notable reliance on the light source was identified, rendering its incorporation imperative for subsequent investigations. Under hypoxic conditions, the bacteria demonstrated adequate oxygen production. The efficacy of cell culture inserts was validated, albeit with a concomitant diminution in the cultivation surface area. 

According to the literature, *Synechococcus* sp. 7002 has hardly been studied in humans. However, due to its increasing use in medicine, it is crucial to clarify the safety of the bacteria’s application. To date, few information has only been published regarding the use of *Synechococcus* sp. PCC 7002. For this purpose, we are the first to examine the effect of the dermal application of cyanobacteria critically. Our present study focuses on investigating the biocompatibility of the bacterial strain *Synechoccous* sp. PCC 7002 with human dermal cells, namely 3T3 fibroblasts and HaCaT keratinocytes. Although no direct cell–cell interaction has been assessed to date, cyanobacterial extracts were tested against primary rat hepatocytes and HL-60 human monocytic leukemia cells, revealing a high percentage of apoptotic cells, which requires a closer look at the application of the bacterial strain [43].

However, to date, there has been no study to demonstrate a direct cell interaction with human skin cells, necessitating further analysis by coculturing the bacteria with human dermal cells. We demonstrated that cyanobacteria can be cultivated alongside both 3T3 fibroblasts and HaCaT keratinocytes without inducing significant growth-inhibiting effects. The investigation delved into the impact on metabolic activity, cell count, and lactate dehydrogenase (LDH) concentration. Initial experiments were performed with 3T3 fibroblasts, revealing a reduction in growth rate compared to the positive control (3T3 monoculture), attributable to the dilution of the culture medium during the production of cocultures. This effect was subsequently rectified and duly considered in subsequent experiments. First, we investigated the possible adverse effects of the AD-7 medium used for bacterial growth on 3T3 cell viability. The metabolic activity revealed no significant differences when the media were diluted 1:1 with AD-7 compared to the cultivation with a regular cell medium after 4 days. Next, we evaluated the effect of coculturing 3T3 with microorganisms. Microscopic imagery depicting the cohabitation of both organisms served as substantiation of their compatibility. A direct positive correlation between the bacterial population and the proliferation rate of fibroblasts was observed, suggesting that cyanobacteria create a conducive growth environment for fibroblasts. Cocultivation with an augmented quantity of bacteria (ratios of 1:5 or 1:25) led to an increase in the metabolic cell activity of 3T3 fibroblasts. In line with this, Toucheteau et al. revealed the stimulating effects of Exopolysaccharides (EPS) from microalgae regarding the collagen synthesis of human fibroblasts, promising a symbiotic effect [44]. In contrast, Yin et al. [45] identified inhibitory effects on the proliferation of both cell types when cyanobacteria were incubated with keratinocytes and fibroblasts. However, they noted stimulatory effects on the migration of keratinocytes in the scratch assay. These findings diverge from the in vitro results presented in this study, as well as their in vivo findings. It should be emphasized that their study was performed on rats and not with human cells, which limits the comparability. The symbiotic effect of cyanobacteria is also supported by the study by Shinohara et al. that proved the growth-promoting effects on human myeloma cell lines of a non-dialyzable extract of *Synechococcus elongatus*, which indicates the promising effects of the cocultivation of cyanobacteria with human cells [46]. Feng et al. were among the first to investigate the influence of bacteria on human cells. They revealed that biogenic polyphosphate nanoparticles from *Synechococcus* sp. PCC 7002 showed intestinal protective potential in human intestinal epithelial cells in vitro and in mouse small intestine ex vivo, supporting biocompatibility with human dermal cells and arguing against the cytotoxicity of the bacteria [47]. To preclude cytotoxic effects in this study, the lactate dehydrogenase concentration was measured, revealing possible cytotoxicity after 2 days, whereas the LDH trend showed a decrease after 4 days compared to the 3T3 monoculture (positive control). The high LDH level after 2 days compared to the 3T3 monoculture may also be due to an enzymatic interaction of the bacteria and needs further investigation. The lactate dehydrogenase concentration exhibited a decrease in cocultures over the 4-day period, in contrast to a notable increase observed in the positive control group. Concurrently, a consistent growth in the cell count over four days was detected without significant differences to the 3T3 monoculture. Taken together, the cyanobacteria had no cytotoxic effects on 3T3 fibroblasts after 4 days, whereas coculturing with microorganisms led, on the one hand, to a stimulating effect on the metabolic activity of 3T3 and, on the other hand, to an increased LDH level after 2 days.

Besides fibroblasts, keratinocytes have a crucial role in the development of an intact skin barrier. Consequently, the exclusion of the possible adverse influences of cyanobacteria on these cells through cocultivation is imperative. Li et al. focused on the impact on the intestinal epithelial barrier. They investigated the cytotoxicity of cyanobacteria on the epithelial cells of the mammalian interstitium in animal experiments. Their safety assessment of the cyanobacterial strain utilized in this study revealed no acute toxicity or mortality after administering the bacteria to mice via gavage at a dose of 10 g per kg body weight. Furthermore, in the 90-day subchronic toxicity study conducted with mice, no treatment-related mortality or clinical symptoms were observed, and there were no adverse effects noted [48]. According to this, the contact of cyanobacteria with epithelial cells of the mammalian interstitium has already been successfully tested in vivo. To date, there are no data on the application of the bacteria on our skin barrier. For this purpose, the cyanobacteria were cocultivated with human keratinocytes, as already described. We measured a reduced metabolic activity after two days compared to the positive control. These findings were not sustained after four days of incubation. The DNA content was determined after two and four days to ascertain the proliferation capacity of HaCaT with precision, revealing no significant proliferation losses attributable to the supplemented bacteria. Taken together, in the case of keratinocytes, no disparities in the metabolic activity or DNA concentration were discerned after a span of four days. These findings contradict the hypothesis of incompatibility between cyanobacteria and HaCaT keratinocytes. According to the well-established wound healing process, a heightened keratinocyte–fibroblast interaction and their growth factor networks stimulate the proliferation and migration of both cell types, contributing to granulation tissue formation [49]. Additionally, Williams et al. [50] demonstrated a significant upregulation of the keratinocyte chemoattractant/growth-regulated oncogene (KC/GRO) just four hours after the intravenous injection of *Synechococcus elongatus* in rats. This genetic basis suggests a stimulation of proliferation in this cell line. While these investigations were performed on rats and cannot be directly extrapolated to humans, the results align with the in vitro findings presented in this paper, indicating a high tolerance for cyanobacteria. Nonetheless, assertions regarding potential long-term effects are precluded due to the brief incubation period.

In summary, we contributed to the proof of biocompatibility of *Synechococcus* sp. PCC 7002 with human dermal cells by coculturing. Despite occasional conflicting results in the literature, no adverse effects have been observed to date. Our results unequivocally indicate that cyanobacteria do not exert detrimental effects on human dermal cells, such as 3T3 fibroblasts and HaCaT keratinocytes. As a component of our preliminary investigations, we have already demonstrated the feasibility of coculturing cyanobacteria with lymphatic endothelial cells [26]. Consequently, cyanobacteria exhibit high biocompatibility and hold considerable promise for integration into biomedical approaches for tissue regeneration, especially for chronic wounds. However, further in vivo studies are necessary to ensure the safe use of cyanobacteria in humans.

## 4. Materials and Methods

### 4.1. Cell Culture of Cyanobacteria

Transgenic *Synechococcus* sp. PCC 7002 (SynHA12) were cultivated in A-D7 medium agar plates supplemented with glucose (1 g/L) and chloramphenicol (10 μg/mL) at 30–50 μE m^−1^·s^−1^ and 25–30 °C [13]. The plates were refreshed every 3 weeks. The strain SynHA12 was used for all experiments since their genetic antibiotic resistance facilitated sterile culture conditions and prevented contamination. A liquid preculture was started before each experiment by inoculating agar-growing cyanobacteria in 50 mL A-D7 medium (supplemented with 1 g/L glucose) and incubating it for 3 days at the standard bacterial culture conditions (30 °C, 150 rpm, 30–50 μE m^−1^·s^−1^). For all cell-culture experiments, the cyanobacteria cell number was determined by light microscopy (Primovert, Zeiss, Oberkochen, Germany) using a Neubauer cell chamber. The cyanobacteria were then cultured at 20 °C, 30 °C, and 37 °C according to the experimental setup; 5% CO_2_; and constant illumination by placing them under a light source with the complete spectrum of white light at a distance of 25 cm above the samples (32.25 μE m ^−1^·s ^−1^, LED, Sebson, Dortmund, Germany) to allow photosynthetic growth. For the detection of light dependency, the cells were incubated under lighting conditions and in the absence of a light source according to the experimental setup.

### 4.2. Bacterial Proliferation

To assess the bacterial proliferation capacity, bacteria were seeded at 1 × 10^6^ in 12-well plates without human cells. The cyanobacteria cell number was determined by light microscopy (Primovert, Zeiss, Oberkochen, Germany) using a Neubauer cell chamber. To detect the impact of the cell culture inserts, the bacteria were cultured either with or without cell culture inserts (0.4 µm pore size) as a positive control. The impact of illumination was investigated under different lighting conditions according to the experimental setup. Furthermore, the bacteria were cultured at different temperature levels according to the experimental setup (20 °C, 30 °C, and 37 °C).

### 4.3. Oxygen Release Measurement

Dissolved oxygen concentrations were measured in Oxodishes (OD24, OD-1842-01, PreSens GmbH, Regensburg, Germany) every 10 min using the SensorDishReader system (PreSens GmbH, Regensburg, Germany), according to the manufacturer’s instructions. This system monitors the percentage of dissolved oxygen content (% pO_2_) in the culture medium using a fluorometric oxygen sensor. Cocultures with different bacterial concentrations were prepared as described previously and cultured for 3 days under hypoxic conditions (<1% pO_2_) with constant illumination. The control group contained regular AD-7 medium in the absence of the cyanobacteria.

### 4.4. Cell Culture of 3T3 Fibroblasts and HaCaT Keratinocytes

HaCaTs (human adult low-calcium high-temperature keratinocytes) were regularly purchased from PromoCell (Heidelberg, Germany). They are classified as non-tumorigenic despite their immortality allowing over 100 passages. The fibroblasts were kindly provided by the Biomedical Center within the Faculty of Biology at LMU University (Professor Nickelsen). Both eukaryotic cell types were cultured in DMEM (Biochrom GmbH, Berlin, Germany) with 10% fetal bovine serum (FBS) at 37 °C in a standard mammalian incubator (HERACell Vios 160i) with 5% CO_2_ and a humidity of 90%. The medium was supplemented with 1% penicillin–streptomycin and 1% amphotericin B to avoid contamination. The medium was changed every 2–3 days.

### 4.5. Coculture of Human Cells and Cyanobacteria

**Figure 4:**
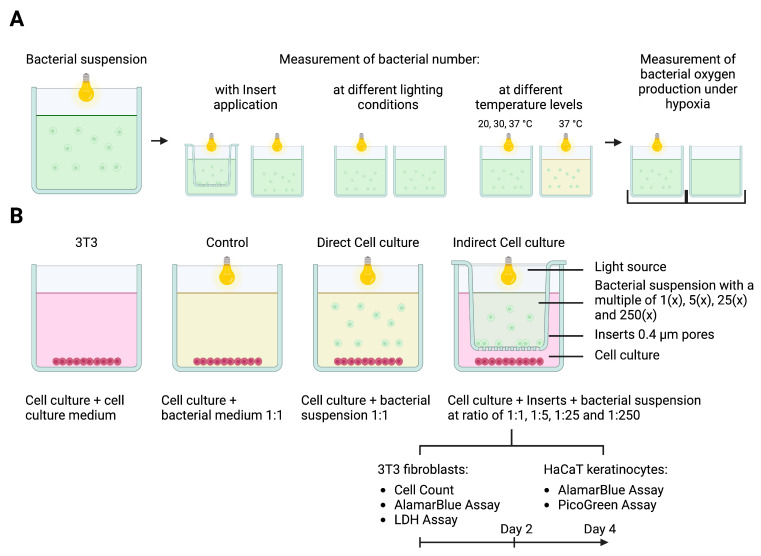
(**A**) Experimental setup for the adaptation of cyanobacteria to human cell culture conditions. Schematic illustration of the measurement of the bacterial number with insert application, as well as at different lighting and temperature levels. Furthermore, we assessed the bacterial oxygen production under hypoxic conditions. (**B**) Schematic illustration of direct and indirect coculturing with cell culture inserts—created with BioRender.com, accessed on 11 February 2024: Direct cocultures allow cell–cell contact. In comparison, the use of cell culture inserts enables regular medium and oxygen exchange between the bacterial suspension and the cell culture via a membrane in the culture medium with 0.4 μm pores. The inserts are immersed approx. 0.2 cm into the culture medium. The light source enables continuous oxygen production through photosynthesis by the microorganisms.

The cocultivation of different cell lines with cyanobacteria was performed either with direct cell contact or indirectly by the spatial separation of the two organisms. Indirect cocultures of human cells and cyanobacteria were established using 0.4 μm pore cell culture inserts (Greiner Bio-One, Kremsmünster, Austria), which allowed the sharing of the media and oxygen diffusion but provided spatial separation between the two different cell types. The experimental setup is shown schematically in Figure 4.

3T3 fibroblasts were seeded at a density of 2 × 10^4^ per cm^2^ in an Oxodish OD 24-well plate (PreSens) and supplemented with a cyanobacteria count at a ratio of 1:5 (1×), 1:5 (5×), 1:25 (25×), or 1:250 (25×), depending on the experimental condition. A total of 1.5 × 10^4^ keratinocytes per cm^2^ were cocultured with cyanobacteria at a ratio of 1:250 (250×) in an Oxodish OD 24-well plate (PreSens). For this purpose, 500 µL A-D7 medium of the bacterial suspension was diluted with 500 µL DMEM supplemented with 20% FBS in each well. For the constant monitoring of photosynthetically produced oxygen, cells were seeded in Oxodish OD 24-well plates (PreSens) at the appropriate density for each cell type followed by incubation in antibiotic-free cell-specific medium for 8 h to allow the cells to adhere to the plate bottom. All cocultures were supplemented with 0.1% chloramphenicol (Sigma-Aldrich, Taufkirchen, Germany) to protect against contamination and with 25 mM/mL HEPES (Sigma-Aldrich) for pH stabilization.

The designed cocultures were cultivated in a standard incubator at 37 °C with 5% CO_2_ and constant illumination (Sebson, LED) to stimulate photosynthesis, depending on the experimental conditions. The cultivation period lasted 1, 2, 4, or 7 days, depending on the experiment. As a control for HaCaT, the respective cell type was cultivated in the absence of cyanobacteria in the same dilution ratio of the specific cell medium in A-D7 medium. The positive control group, consisting of 3T3 fibroblasts, underwent no adaptation of the cell medium and was not diluted with AD-7 medium. The negative control group (control) was established in the absence of cyanobacteria, yet adhered to a 1:1 dilution ratio with the bacterial medium. All experiments were performed in triplicate, and the medium was changed after each measurement.

Pictures were taken using a brightfield microscope (Axio Observer, Zeiss, Ober-kochen, Germany), and the numbers of cells were determined by blinded digital image analysis using ImageJ Cell-counter (ImageJ V-1.52A [51]). To determine the number of cyanobacteria in each well, the bacteria were resuspended several times and counted using the Neubauer cell chamber, as described before.

### 4.6. 3T3 Fibroblast Proliferation

To detect the 3T3 fibroblast proliferation capacity, 2 × 10^4^ cells per cm^2^ were seeded in 24-well plates. The cell number was determined using Countess 2 FL (Invitrogen, Carlsbad, CA, USA). A cell count analysis was performed at a 1:1 dilution of the DMEM cell medium with A-D7 medium in bacterial cocultures compared to a control group in the absence of the bacteria (control) and 3T3 pure culture (3T3) over a 4-day period.

### 4.7. AlamarBlue Assay

For the quantification of the metabolic activity of human cells, the AlamarBlue assay (AlamarBlue Cell Viability Reagent, Invitrogen, Carlsbad, CA, USA) was performed, according to the manufacturer’s instructions. First, cells were seeded in triplicate in a 24-well plate at a density of 2 × 10^4^ cells/cm^2^ for fibroblasts and 1.5 × 10^4^ cells/cm^2^ for keratinocytes and allowed to attach for 2 h. Then, bacteria were added and cocultured, as described previously. At each experimental time point, the cells were washed with PBS (Gibco TM, Thermo Fischer Scientific, Waltham, MA, USA) to avoid possible bacterial residues and incubated for 2 h in an AlamarBlue working solution (0.1 mg/mL in DMEM without phenol red) under standard culture conditions. Triplicates of the AlamarBlue working solution were prepared as blank samples and treated under the same conditions. After incubation, 100 μL aliquots of the suspension were transferred to a 96-well plate, and cell viability was determined using a fluorescence plate reader (Infinite M Plex, TECAN, Männedorf, Switzerland) at Ex./Em. 560 nm/590 nm. After measurement, the AlamarBlue working solution was replaced by a medium suitable for the respective experiment for further incubation.

### 4.8. PicoGreen Assay

The assay was performed according to the manufacturer’s instructions (Quant-iTTM PicoGreenTM dsDNA Reagent and Kit, Invitrogen Thermo Fisher Scientific). First, keratinocytes were cultured at a density of 1.5 × 10^4^ per cm^2^ in a 24-well plate in triplicate for 2 to 4 days. After the respective experimental duration, the cells were detached from the bottom and collected in a 2 mL Eppendorf tube. The samples were centrifuged at 5000× *g* for 5 min (Eppendorf Centrifuge 5415 C), and the supernatant was discarded. The resulting cell pellet was stored in aliquots at −20 °C. To create a standard curve, we prepared a stock solution with a concentration of 2 µg/mL dsDNA. From the initial sample, 500 µL was transferred to a second Eppendorf tube with 500 µL distilled water already added and repeatedly resuspended. This step was repeated twelve times, continuing the dilution series to a concentration of 1.95 ng/mL. A blank value was represented by a sample with pure distilled water. The frozen cell pellet was thawed at room temperature, and 100 µL Proteinase K working solution, along with a 7 mm diameter stainless steel bead, was added. The mixture was dissolved for 10 min at 50 Hz in a TissueLyser LT (Qiagen, Hilden, Germany). An additional 900 µL of Proteinase K working solution was added, and the cells were lysed overnight at 56 °C and 1000 rpm in a Thermocycler (Eppendorf, Hamburg, Germany). Lysis was stopped at −20 °C, and the samples were centrifuged at 6000 rpm for 5 min (Eppendorf Centrifuge 5415 C). We transferred 75 µL of the supernatant to a new 1.5 mL Eppendorf tube already containing 225 µL TE buffer. After vortexing the sample solution several times (Heidolph Reax Top, Schwabach, Germany), we pipetted 100 µL into a black 96-well plate with an additional 100 µL PicoGreen dye solution. The samples of the standard curve were treated similarly and mixed with the dye solution. The incubation of the samples occurred in the dark for 5 min at room temperature. Subsequently, the absorbance and fluorescence emission at a wavelength of 504 nm/550 nm (Ex./Em.) were measured using a fluorescence photometer (Infinite M Plex, TECAN). A linear standard curve was built using the values of the dilution series, which served to determine the DNA content of the samples under investigation. Finally, the determined fluorescence values were presented as the DNA quantity in ng/mL of the samples.

### 4.9. Lactate Dehydrogenase Assay

To investigate cytotoxicity, the released lactate dehydrogenase (LDH) in the culture medium was quantified. Cytotoxicity is directly proportional to the measured LDH concentration in the cell culture media [52]. The assay was performed according to the manufacturer’s instructions (LDH-Cytotoxicity Assay Kit II, Sigma-Aldrich, Taufkirchen, Germany). For the LDH assay, 2 × 10^4^ 3T3 fibroblasts were cocultivated directly and indirectly with *Synechococcus* sp. PCC 7002 in a 24-well plate, as described previously. First, for the assessment of the toxic potential of the pure A-D7 medium of cyanobacteria on human cells, 3T3 fibroblasts were cultivated in DMEM diluted 1:1 in A-D7 medium in triplicate for 4 days. To investigate the impact of cyanobacteria on cells, we prepared a coculture with different ratios of cyanobacteria (1:5, 1:25), as described previously. The coculture was incubated at 37 °C in a 5% CO_2_ atmosphere for 2 and 4 days; the medium was collected and centrifuged at 300× *g* for 5 min; and the supernatant was aspirated, transferred to a 2 mL Eppendorf tube, and temporarily stored at 4 °C. Prior to the assay execution, the samples were thawed and resuspended. A total of 50 µL was pipetted into a transparent 96-well plate in technical triplicates. As a control, the same 1:1 dilution of both media was used but without prior use in a cell culture. To each sample, 50 µL of LDH assay solution (Invitrogen™) was added using an electronic pipette (Easypet, Eppendorf) and incubated in the absence of light for 30 min at room temperature. Subsequently, the absorption was photometrically measured at a wavelength of 490 nm and a reference wavelength of 690 nm (Plate Reader Infinite M Plex, TECAN). The mean values were calculated and graphically represented.

### 4.10. Statistical Analysis and Illustrations

All assays were performed in at least 3 independent experiments with at least 3 technical replicates in each experimental group. All data are presented as the mean and standard deviation. A Student’s *t*-test was performed for comparison between the two different groups. Differences among groups were considered significant if *p* ≤ 0.05 (ns: not significant; * *p* ≤ 0.05; ** *p* ≤ 0.01; and *** *p* ≤ 0.001). All data were tested for normal distribution before performing a *t*-test. Schematic representations were created using the platform www.BioRender.com, accessed on 11 February 2024.

## 5. Conclusions

In conclusion, cyanobacteria are easily adaptable to human cell conditions, exhibiting no proliferation constraints. These bacteria demonstrate a high capacity for photosynthetic oxygen provision under constant illumination, contingent upon their bacterial abundance. The inherent reliance on light for photosynthesis underscores their substantial potential for diverse biomedical applications, particularly on the external surface of the human body.

In pursuit of this potential, the study substantiates the biocompatibility of cyanobacteria and their associated culture medium with HaCaT keratinocytes and 3T3 fibroblasts by successful cocultivation. This study serves as a foundational step, thereby opening possibilities for subsequent tissue engineering experiments, with the ultimate goal of application to human skin, especially in chronic wounds. However, comprehensive in vivo studies are imperative to ascertain and mitigate potential systemic reactions that may arise in response to the introduction of cyanobacteria in such contexts.

## Data Availability

The data presented in this study are available on request from the corresponding author.

## Abbreviations

DMEM	Dulbecco′s modified Eagle′s medium
DNA	Deoxyribonucleic acid
FBS	Fetal bovine serum
HaCaT	Human adult low-calcium high-temperature keratinocytes
HEPES	Hydroxy-ethyl-piperazine ethane sulfonic acid
LDH	Lactate dehydrogenase
PBS	Phosphate-buffered saline
